# Expression of Amphiregulin in Enchondromas and Central Chondrosarcomas

**DOI:** 10.6061/clinics/2021/e2914

**Published:** 2021-08-16

**Authors:** Daniele Moraes Losada, André Luiz da Cruz Ribeiro, Francisco Fontes Cintra, Guilherme Rossi Assis de Mendonça, Maurício Etchebehere, Eliane Maria Ingrid Amstalden

**Affiliations:** I Departamento de Patologia, Universidade Estadual de Campinas (UNICAMP), Campinas, SP, BR.; II Departamento de Ortopedia e Traumatologia, Universidade Estadual de Campinas (UNICAMP), Campinas, SP, BR.

**Keywords:** Bone Tumors, Amphiregulin, Enchondroma, Chondrosarcoma, Immunohistochemistry

## Abstract

**OBJECTIVES:**

The aim of this study was to evaluate the role of amphiregulin protein, an epidermal growth factor receptor ligand, in cartilaginous tumors.

**METHODS:**

Amphiregulin expression was examined in 31 enchondromas and 67 chondrosarcomas using immunohistochemistry analysis.

**RESULTS:**

Overall, 15 enchondromas (48.40%) and 24 chondrosarcomas (35.82%) were positive for amphiregulin. According to the receiver operating characteristic curve test, no difference in amphiregulin expression was observed between enchondromas and low-grade chondrosarcomas (*p*=0.0880). Additionally, 39 lesions (16 in short bones, 13 in long bones, and 10 in flat bones) were positive for amphiregulin, exhibiting a higher percentage of positive cells (*p*=0.0030) and intensity of immunohistochemical expression (*p*=0.0055) in short bone lesions than in others. Among 25 enchondromas localized in short bones, 15 expressed amphiregulin; however, all 6 cases localized in long bones were negative for this marker (*p*=0.0177).

**CONCLUSIONS:**

Amphiregulin did not help in distinguishing enchondromas from low-grade chondrosarcomas. The present study is the first to document the expression of this immunohistochemical marker in enchondromas. Furthermore, amphiregulin expression in enchondromas was localized in short bones, indicating a phenotypic distinction from that in long bones. This distinction may contribute to an improved understanding of the pathogenesis of these lesions.

## INTRODUCTION

Enchondroma (ENC) is a benign cartilaginous tumor located in the bone marrow cavity, accounting for 3.00-10.00% of primary bone tumors. Histologically, it is a hypocellular lesion with an abundant hyaline cartilaginous matrix, small round nuclei, and condensed chromatin. However, when located in short tubular bones, ENC may exhibit a more myxoid stroma and be highly cellular, with open nuclear chromatin containing a small nucleolus. Regardless of its location, no lamellar bone permeation, cortical bone destruction, or soft tissue extension has been documented. The presence of these findings favors the diagnosis of chondrosarcoma (1,2).

Notably, chondrosarcoma is a malignant cartilaginous tumor that accounts for 25.00% of all primary bone sarcomas. Approximately 90.00% of these tumors constitute a group of central chondrosarcomas (CCS), which are histologically classified into low-grade (atypical cartilaginous tumor/grade 1 chondrosarcoma; LGC), intermediate-grade (grade 2 chondrosarcoma; IGC), and high-grade (grade 3 chondrosarcoma; HGC). This histological graduation considers the following parameters: nuclear size, hyperchromatism, cellularity, and mitotic index (1,3). In this group of neoplasms, the frequency of adverse events (recurrence, metastasis, and death) is directly related to tumor progression; therefore, high-grade chondrosarcomas are the most aggressive and prone to metastases (2,4).

The histological distinction between ENC and LGC can be difficult and is subject to substantial interobserver variations, thus requiring collaborative evaluation using clinical and radiological data. However, the Kappa coefficient of agreement between pathologists reaches only a modest value of 0.54 (5). In this setting, there are five parameters that, if combined, can aid in differentiation: high cellularity, bone permeation, open chromatin, myxoid degeneration of the matrix exceeding 20.00%, and age of the patient over 45 years (1). In lesions located in the short tubular bones, the main discriminatory morphological parameters include the presence of mitosis, rupture of the bone cortex, permeation of bone trabeculae, and involvement of soft tissues (1).

Several oncogenic signaling pathways have been implicated in the tumorigenesis and/or progression of cartilaginous neoplasms. In this context, the potential role of amphiregulin should be considered, as this epidermal growth factor receptor (EGFR) ligand physiologically acts on normal bone development and contributes to adequate cell migration by upregulating the expression of integrins (heterodimeric transmembrane glycoprotein family that facilitates cell-cell and cell-matrix interactions) (4,6-8). In addition to this crucial physiological role, several studies have demonstrated its role as a predictive marker for metastases and tumor progression (4,9-12). Although a previous study (4) has associated amphiregulin with tumor progression in chondrosarcomas, the immunoexpression of this antibody in ENC and its role in distinguishing ENC from LGC remain unclear. Herein, we aimed to evaluate the immunoexpression of amphiregulin in ENC and CCS and associate this expression with histological grade and clinical and prognostic data.

## METHODS

### Ethical Approval

All procedures performed in this study, including the use of specimens harvested from human subjects, were in accordance with the ethical standards of the institutional and national research committees (HC-Unicamp, ref: 02184318.6.0000.5404, approved on December 4, 2018) and with the 1964 Helsinki declaration and its later amendments or comparable ethical standards.

### Patient and Tumor Samples

In total, 98 patients (37 men and 61 women, aged 9 to 87 years) diagnosed with ENC or CCS using biopsy and/or resection specimens were identified at the Department of Pathology at the University Hospital of the State University of Campinas from 1994 to 2019. The tumor specimens were fixed in 10.00% formalin and later decalcified with hydrochloric acid and ethylenediaminetetraacetic acid. The samples consisted of 31 ENC and 67 CCS, including 28 LGC, 31 IGC, and 8 HGC, according to the World Health Organization 2020 classification system (Table 1) (1). Clinical data (age, sex, location, type of bone affected, and follow-up information) were obtained by reviewing the patients’ medical records and were considered favorable in the absence of adverse events (death, recurrence, or metastasis) or unfavorable in the presence of at least one adverse event. No patient had received any treatment before surgery. All patients with ENC and LGC underwent curettage, and all patients with IGC and HGC underwent *en bloc* resection. Representative formalin-fixed paraffin-embedded blocks were selected for immunohistochemical analysis.

### Immunohistochemical Technique and Analysis

**Figure 1:**
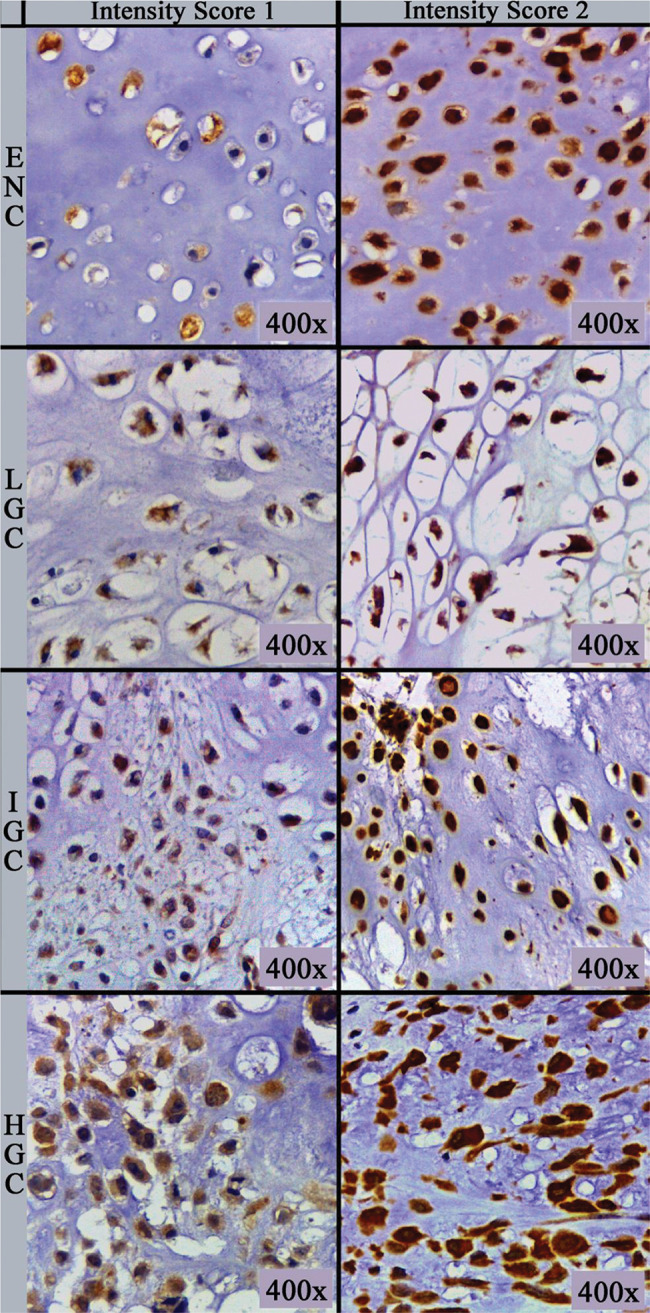
Intensity score by diagnostic group. Antibody: Amphiregulin (original magnification, 400×). ENC, enchondroma; LGC, low-grade chondrosarcoma; IGC, intermediate-grade chondrosarcoma; HGC, high-grade chondrosarcoma.

The primary antibody (clone sc-74501; Biotech. Inc., Santa Cruz, CA) used was a monoclonal mouse antibody against amphiregulin at a dilution of 1:100 in phosphate-buffered saline supplemented with 1.00% bovine serum albumin. Immunohistochemical staining was performed on 5 µm thick sections processed from formalin-fixed paraffin-embedded tissues, which were mounted on silanized slides, briefly deparaffinized in xylol, and rehydrated in serial alcohol. Epitope retrieval was achieved by steaming with citrate buffer at 95°C. The EnVision+Dual Link System HRP polymer (Dako, Glostrup, Denmark) was used as the reaction amplifier. The antibody complex was visualized with 3,3’-diaminobenzidine tetrahydrochloride (Dako). Then, the sections were counterstained with hematoxylin. Appropriate negative and positive controls were included in each assay according to the manufacturer’s instructions. Assessment of immunohistochemical staining was evaluated by two independent pathologists (DML and EMIA), blinded to the clinicopathological parameters of the patients. Amphiregulin-positive tumor cells showed cytoplasmic immunoreactivity. Immunohistochemical analysis was performed as described by Zhu et al. (13) and is based on the multiplication of scores for intensity of staining and percentage of immunostained cells, resulting in an immunoreactivity score. The number of positively stained cells was photographed (Leica ICC50 HD^®^) in five “hot spots” at the highest magnification (40× objective) microscopic fields, and the percentage of positive cells was calculated using ImageJ (U.S. National Institutes of Health, Bethesda, Maryland, USA, http://imagej.nih.gov/ij/) software cell counter plug-in. If the five hot spot fields failed to demonstrate at least 100 cells, new areas were selected. The percentage score (PS) of immunoreactive tumor cells was classified as follows: 0 (0.00%), 1 (1.00-10.00%), 2 (11.00-50.00%) and 3 (>50.00%). The intensity score (IS) was recorded and stratified as follows: 0 (negative/no expression), 1 (weak/light brown), and 2 (strong/dark brown) (Figure 1). The final immunoreactivity scores (IRS) were obtained for each case by multiplying PS and IS and then classified as negative (value equal to 0), low (values equal to 1, 2, or 3), or high (values equal to 4 or 6).

### Statistical Analysis

Data analysis was performed using the SAS System for Windows version 9.4 (SAS Institute Inc., Cary, NC). Chi-square and Fisher’s exact tests were used to compare categorical variables. The Mann-Whitney U or Kruskal-Wallis tests were used to compare numerical variables; the latter was followed by Dunn’s post-hoc test. A receiver operating characteristic (ROC) curve was calculated to determine amphiregulin sensitivity and specificity to distinguish ENC from LGC using PS values. Survival analyses were performed considering disease-specific survival, which was defined as the time from diagnosis until death or the last follow-up. Survival curves were plotted using the Kaplan-Meier method and compared using the log-rank test. Univariate Cox regression analyses were also performed. All variables with a *p*-value less than 0.10 in the univariate Cox regression were included in the multivariate model with a stepwise selection method to identify independent risk factors associated with survival. Statistical significance was set at *p*<0.05.

## RESULTS

In the present study, we noted differences in the age at diagnosis between patients with ENC (mean 31.84 years) and those with CCS (mean 50.40 years) (*p*<0.0001).

**Figure 2:**
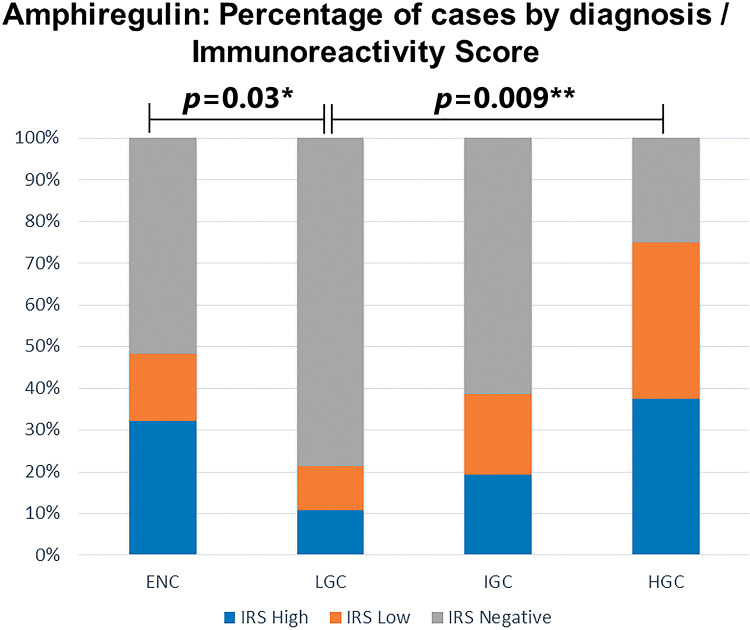
Amphiregulin immunoreactivity score in enchondromas and chondrosarcomas. IRS, immunoreactivity score; ENC, enchondroma; LGC, low-grade chondrosarcoma; IGC, intermediate-grade chondrosarcoma; HGC, high-grade chondrosarcoma. (*) Chi-squared test; (**) Fisher's Exact test.

**Figure 3:**
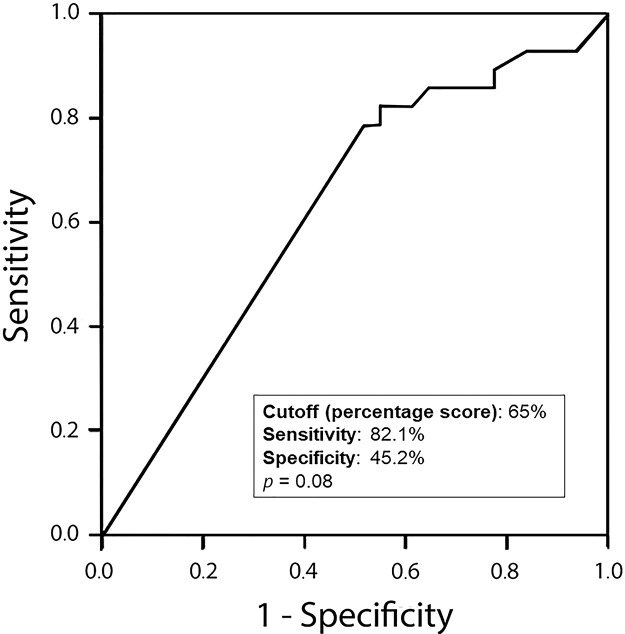
ROC curve of the percentage of positive cells to discriminate enchondromas from low-grade chondrosarcomas. Accuracy, 63.00%; 95% CI, 48.60-77.30; cut-off value, 65.00%; sensitivity, 82.10%; specificity, 45.20%; *p*=0.0880. ROC, receiver operating characteristic; CI, confidence interval.

Amphiregulin immunostaining was positive in 15 of the 31 ENC cases (48.40%), 6 of the 28 LGC cases (21.40%), 12 of the 31 IGC cases (38.80%), and 6 of the 8 HGC cases (75.00%) (Table 2). The analysis of the four groups by Fisher's exact test indicated no significant difference (*p*=0.0916). However, a significant difference in IRS was detected between ENC and LGC (48.40% *vs*. 21.40%, respectively, *p*=0.03; Chi-squared test). In addition, amphiregulin expression was directly associated with the histological grade of chondrosarcomas, with statistical significance observed between low- and high-grade lesions (21.40% *vs*. 75.00%, respectively, *p*=0.009; Fisher's exact test). IRS was low in 17 (17.30%) cases (5 ENC, 3 LGC, 6 IGC, and 3 HGC) and high in 22 (22.40%) cases (10 ENC, 3 LGC, 6 IGC, and 3 HGC) (Figure 2). The ROC curve of the percentage of positive cells for differentiating between ENC and LGC demonstrated an accuracy of 63.00% (95% confidence interval, 48.60-77.30; cut-off value, 65.00%; sensitivity, 82.10%; specificity, 45.20%; *p*-value=0.0880) (Figure 3).

Regarding topography, 26 cases showed localization in short bones (25 ENC and 1 HGC), 53 in long bones (6 ENC, 25 LGC, 18 IGC, and 4 HGC), and 19 in flat bones (3 LGC, 13 IGC, and 3 HGC). Amphiregulin was positive in 16 lesions located in short bones (15 ENC and 1 HGC), 13 in long bones (4 LGC, 7 IGC, and 2 HGC), and 10 in flat bones (2 LGC, 5 IGC, and 3 HGC). IRS was high in 42.30% of lesions located in short bones, 21.05% in flat bones, and 13.21% in long bones (*p*=0.0055). Notably, among ENC cases showing localization in short tubular bones, 15 cases (60.00%) expressed amphiregulin. Conversely, all six cases (100.00%) with localization in long tubular bones were negative for this immunohistochemical marker (*p*=0.0177).

Eighty-three patients presented favorable outcomes, and 15 patients had unfavorable outcomes (recurrence and/or metastasis and/or died of the disease). Until the last follow-up, seven patients showed recurrence, five presented with pulmonary metastasis, and eight had died of the disease. The outcome was favorable in 31 (100.00%) patients with ENC, 27 (96.40%) with LGC, 20 (64.50%) with IGC, and 5 (62.50%) with HGC. Amphiregulin expression was more frequently observed in CCS that evolved with metastasis than in those without metastasis (80.00% *vs*. 37.70%, respectively; *p*=0.0051). No significant difference in amphiregulin expression was observed in patients who exhibited recurrence when compared with that in patients who did not exhibit recurrence (28.60% *vs*. 40.70%, respectively; *p*=0.2404). In addition, no significant difference in the expression of this antibody was observed in patients with fatal outcomes when compared with living patients (50.00% *vs*. 38.90%, respectively; *p*=0.6728).

Differences in the frequency of adverse events were observed between ENC and IGC (0.00% *vs*. 35.50%, respectively; *p*=0.0003), ENC and HGC (0.00% *vs.* 37.50%, respectively; *p*=0.0061), LGC and IGC (3.60% *vs*. 35.50%, respectively; *p*=0.0029), and LGC and HGC (3.60% *vs.* 37.50%, respectively; *p*=0.0278). Considering specific adverse events, differences in the frequency of metastasis outcomes were observed between ENC and HGC (0% *vs*. 25%, respectively; *p*=0.0378) and LGC and HGC (0.00% *vs*. 25.00%, respectively; *p*=0.0444). In addition, we noted differences in the occurrence of death between ENC and IGC (0.00% *vs*. 19.40%, respectively; *p*=0.0240), ENC and HGC (0.00% *vs*. 25.00%, respectively; *p*=0.0378), LGC and IGC (0.00% *vs*. 19.40%, respectively; *p*=0.0240), and LGC and HGC (0.00% *vs*. 25.00%, respectively; *p*=0.0444).

**Figure 4:**
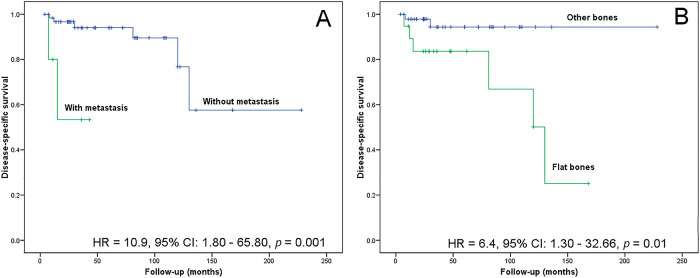
Kaplan-Meier analysis for disease-specific survival. (A) Patients without metastasis show a greater probability of survival than those with metastasis (*p*=0.0010). (B) Patients with tumors located in the appendicular skeleton show a greater probability of survival than those with axial tumors (*p*=0.0100).

The follow-up time for patients with CCS ranged between 4 and 228 months (median, 30 months). The lesion location in flat bones (risk ratio, 6.40; 95% confidence interval, 1.30-32.66; *p=*0.0100) and the metastasis event (risk ratio, 10.90; 95% confidence interval, 1.80-65.80; *p*=0.0010) associated with poor disease-specific survival in CCS (Figure 4). After multivariate analysis, both variables remained independent prognostic factors (risk ratio, 7.43; 95% confidence interval, 1.41-39.18; *p*=0.0181 and risk ratio, 6.99; 95% confidence interval, 1.10-44.50; *p*=0.0395).

## DISCUSSION

ENC and CCS are cartilaginous matrix-producing tumors with distinct biological behaviors. The diagnoses of ENC and CCS have therapeutic consequences ranging from radiological follow-ups to radical surgery (14). Currently, surgical intervention is the treatment of choice, as these tumors demonstrate a limited response to radio and/or chemotherapy treatments (15,16). The histological and radiological similarities between ENC and LGC may impede diagnostic distinction, with large interobserver variations (1). Several research groups have dedicated themselves to identifying complementary diagnostic tools (immunohistochemical markers and molecular methods) that could contribute to an improved understanding of the pathogenesis of these neoplasms, thus enabling therapeutic advances (14,17-21). In this study, the expression of amphiregulin in ENC was analyzed and compared with that in CCS of different histological grades.)

The topography of these neoplasms is an important clinical parameter for differentiating ENC and CCS; cartilaginous lesions involving small bones of the extremities are generally benign, whereas those of flat bones are often aggressive (1,19,20). Although ENC and LGC can be treated with curettage, there is a risk of local recurrence in LGC, approaching 7.0-11.00%, compared with a 0.00% risk in ENC (1,22). The present study revealed the absence of adverse events in ENC; recurrence was observed in a single patient with LGC. There is a risk of malignancy in 1.00% of ENC cases and tumor progression in approximately 10.00% of recurrent LGC cases (1). Herein, no ENC tumors exhibited malignization; the only case of recurrent LGC did not evolve with tumor progression. The worst prognosis was directly associated with the CCS histological grade, similar to findings of previous studies (1,19,20).

Amphiregulin is an EGFR ligand known to be associated with upregulated integrin expression (4,6-8,23). Amphiregulin is overexpressed in several epithelial neoplasms, including prostatic (24), colorectal (12,25), mammary (26), ovarian (27), pancreatic (28), pulmonary (29), hepatic (11,30), and oral (10) neoplasms. Furthermore, amphiregulin is reportedly expressed in mesenchymal tumors and is documented in chondrosarcomas in only four studies (15,16,31,32). In addition, amphiregulin expression has been detected in few other examples of mesenchymal lesions, such as malignant fibrous histiocytoma (33), osteosarcoma (34), and fibrous and osseous dysplasia of the jaw (35,36). The present study demonstrated the cytoplasmic expression of amphiregulin in both ENC and CCS. In CCS, amphiregulin expression was directly proportional to tumor grade; it was expressed in 27.66% of tumors located in the long bones and 52.63% in the flat bones. In ENC, cytoplasmic expression was observed in 60.00% of lesions located in the short bones. The cytoplasmic expression may reflect excessive production, inefficient nuclear translocation, or increased binding and internalization of amphiregulin (37). According to Bostwick et al. (24), the presence of amphiregulin in benign epithelial tumors indicates its role during the early steps of carcinogenesis and can be, at least partly, explained by activating EGFR, which, in turn, induces cell proliferation. We believe that this interpretation can be applied to benign mesenchymal lesions.

The present study demonstrated the cytoplasmic expression of amphiregulin in both ENC and LGC. Although LGC exhibits significantly less amphiregulin expression than ENC, the ROC curve revealed no difference between these two groups. Accordingly, this immunomarker demonstrated no substantial benefit in this diagnostic differentiation. To our knowledge, this is the first study to document amphiregulin expression in ENC. Notably, its positivity was shown to be exclusive in lesions located in short bones and can be a parameter that identifies ENC tumors of this topography, which may have their own clinicopathological features. This study highlights the possibility that tumorigenesis of ENC can employ different signaling pathways depending on the topography, resulting in distinct immunophenotypic characteristics.

In bone-cartilaginous mesenchymal tissue, the important physiological role of amphiregulin in bone formation process (6) and its prognostic role in malignant cartilaginous neoplasms (31) have been previously documented. Furthermore, several studies have shown that increased expression level and signaling of integrins in chondrosarcomas can be related to the migration, invasion, and metastasis of tumor cells (6,38,39). In the present study, we detected significantly upregulated amphiregulin expression in patients with CCS that evolved with tumor metastasis. This finding can be, at least partly, explained by the role of amphiregulin in increasing cellular integrins, disrupting cell-cell and cell-matrix interactions. Therefore, our findings corroborate the hypothesis of Huang et al. (31) that amphiregulin may be considered a potential therapeutic target for treating metastatic CCS.

To our knowledge, this is the first study to present the immunoexpression of amphiregulin in ENC, particularly in the acral bones. The positivity of this immunomarker in this topography can contribute to comprehensively clarifying the pathogenesis of cartilaginous tumors located in the short bones of the appendicular skeleton.

## AUTHOR CONTRIBUTIONS

Losada DM was responsible for the study conceptualization, methodology, validation, investigation, data curation, manuscript drafting, writing, editing and review, and project administration. Ribeiro ALC was responsible for the investigation and data curation. Cintra FF and Etchebehere M were responsible for the conceptualization, methodology and resources. Mendonça GRA was responsible for the formal analysis, manuscript drafting, writing, editing and review. Amstalden EMI was responsible for the conceptualization, methodology, validation, investigation, resources, manuscript writing, editing and review supervision, and funding acquisition.

## Figures and Tables

**Table 1:** Histopathological criteria for enchondroma and central chondrosarcomas according to the World Health Organization Classification of Tumors of Soft Tissue and Bone, 2020.

	Histopathological criteria
Enchondroma	Multinodular or confluent architecture; lobulated growth pattern with typical deposition of bone surrounding the tumor lobules; abundant hyaline cartilage matrix; low cellularity; nuclei generally small, condensed, and uniform in size, with no cytological atypia or mitoses. In small phalangeal bones: more cellularity; occasionally more-open chromatin and small nucleoli; matrix can be myxoid.
Central atypical cartilaginous tumor/Chondrosarcoma, grade 1 (ACT/CS1)	Lobulated growth with typical encasement pattern; lobules can be irregularly shaped and vary in size; abundant hyaline cartilage matrix; low cellularity but slightly higher than in enchondroma; nuclei generally small, condensed, and uniform in size. Binucleation can be seen, and mitosis is absent. Lobular growth patterns can cause cortical thinning and can occasionally permeate and entrap pre-existing lamellar bone trabeculae.
Central chondrosarcoma, grade 2	Lobular configuration with evident permeation and entrapment of pre-existing bone trabeculae; hyaline cartilage matrix with variable myxoid changes; higher cellularity than in ACT/CS1; nuclei can vary in size and display open chromatin and a visible nucleolus; mitosis is present.
Central chondrosarcoma, grade 3	Hyaline cartilage matrix with wide myxoid areas; lobules exhibit spindled and fewer differentiated cells; high cellularity with nuclear atypia and pleomorphism; mitosis is easily observed. Marked entrapment of pre-existing lamellar bone with cortex destruction.

**Table 2:** Amphiregulin expression status by topography.

Neoplasia	Short tubular bone		Long tubular bone		Flat bone		Total no. (%)	*p*-value*
	Pos. no. (%)	Neg. no. (%)	Pos. no. (%)	Neg. no. (%)	Pos. no. (%)	Neg. no. (%)		
ENC	15 (48.40%)	10 (32.30%)	0 (0.00%)	6 (19.30%)	0 (0.00%)	0 (0.00%)	31 (100.00%)	0.0177^a^
LGC	0 (0.00%)	0 (0.00%)	4 (14.20%)	21 (75.00%)	2 (7.20%)	1 (3.60%)	28 (100.00%)	0.1068^b^
IGC	0 (0.00%)	0 (0.00%)	7 (22.60%)	11 (35.50%)	5 (16.10%)	8 (25.80%)	31 (100.00%)	1.0000^b^
HGC	1 (12.50%)	0 (0.00%)	2 (25.00%)	2 (25.00%)	3 (37.50%)	0 (0.00%)	8 (100.00%)	0.4286^b^; 1.0000^a,c^

ENC, enchondroma; LGC, low-grade chondrosarcoma; IGC, intermediate-grade chondrosarcoma; HGC, high-grade chondrosarcoma; Neg. no, negative number; Pos. no, positive number. ^a^=short tubular bone *vs*. long tubular bone; ^b^=long tubular bone *vs*. flat bone; ^c^=short tubular bone *vs*. flat bone. *Fisher's exact test.
