# Erratum for: Expression of Amphiregulin in Enchondromas and Central Chondrosarcomas

**DOI:** 10.6061/clinics/2021/e2914err

**Published:** 2021-11-24

CLINICS (Sao Paulo). 2021;76:e2914err

Erratum for: doi: https://doi.org/10.6061/clinics/2021/e2914, published in 2021.

In the article **Expression of Amphiregulin in Enchondromas and Central Chondrosarcomas**, RESULTS section in the Abstract, remove the following sentences:

“Enchondromas or chondrosarcomas? Please clarify. This will help understand the next sentence on enchondromas localized in short bones and long bones.”

